# Dynamic 3D microfluidic platform for exploring combined targeted therapy, chemotherapy, and virotherapy delivery in ovarian cancer

**DOI:** 10.1007/s13346-025-01997-4

**Published:** 2025-11-03

**Authors:** Lukasz Kuryk, Sara Mathlouthi, Lisa Casagrande, Cristiano Pesce, Francesco Tognetti, Alessio Malfanti, Aleksander Masny, Paolo Caliceti, Mariangela Garofalo

**Affiliations:** 1 Department of Pharmaceutical and Pharmacological Sciences, University of Padua, Via F. Marzolo 5, 35131 Padua, Italy; 2 Department of Virology, National Institute of Public Health NIH - National Research Institute, Chocimska 24, 00-791 Warsaw, Poland

**Keywords:** Ovarian cancer, Microfluidics, 3D tumor model, Oncolytic adenovirus, Drug delivery, Immunogenic cell death

## Abstract

Ovarian cancer poses a persistent therapeutic challenge due to late-stage diagnosis, frequent relapse, and resistance to standard therapies. While oncolytic viruses (OVs) offer a promising immunotherapeutic approach, their clinical efficacy remains limited by an immunosuppressive tumor microenvironment (TME) and inefficient delivery. To address these barriers, we developed a dynamic microfluidic-based 3D ex vivo tumor model to evaluate a systemic, multimodal treatment strategy in ovarian cancer. The model incorporates perfusable tumor spheroids cocultured with peripheral blood mononuclear cells (PBMCs) and endothelial cells (HUVECs), enabling the simulation of vascularized tumor environments and systemic drug perfusion. All therapeutic agents—including the oncolytic adenovirus Ad5/3-D24-ICOSL-CD40L, cisplatin, paclitaxel, and nintedanib—were administered through flow-based circulation to more accurately replicate human pharmacokinetic conditions and tumor-drug interactions. Our results demonstrated that a priming regimen—where Ad5/3-D24-ICOSL-CD40L was administered 48 h before chemotherapy—significantly outperformed the co-administration strategy, reducing spheroid areas and mitigating tumor rebound. Enhanced therapeutic response was associated with increased viral replication, sustained immunogenic cell death, and improved immune cell infiltration, underscoring the importance of sequencing and microenvironment preconditioning. This tumor-on-a-chip platform provides a physiologically relevant tool for real-time monitoring of treatment response, immune activation, and drug delivery under continuous flow. By bridging the gap between traditional in vitro models and in vivo studies, it offers a powerful preclinical system for optimizing combination regimens and advancing personalized therapies in ovarian cancer.

**Graphical abstract:**

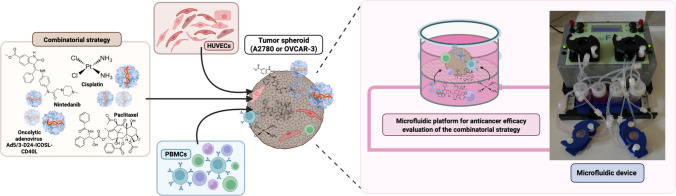

**Supplementary Information:**

The online version contains supplementary material available at 10.1007/s13346-025-01997-4.

## Introduction

Ovarian cancer, often referred to as "silent killer" due to its heterogeneous nature and limited therapeutic options, poses a considerable global health challenge, characterized by concerning rates of incidence and mortality [1, 2]. According to GLOBOSCAN 2022 the global incidence of ovarian cancer was 6.7 cases per 100.000 women, accompanied by a mortality rate of 4.0 per 100.000 women. Projections indicate that by 2050, the number of ovarian cancer cases will increase by over 55%, reaching approximately 503.448 cases. Likewise, the number of deaths is expected to rise by nearly 70% compared to 2022 [3]. The prognosis is generally unfavorable, primarily because accurate diagnosis often occurs only in the later and more advanced stages, mainly due to the vague and commonly seen clinical symptoms [4]. Statistics reveal that over 70% of ovarian cancer cases are diagnosed at stage III or IV, and the five-year survival rate for affected patients is estimated to be approximately 47% [5]. Epithelial ovarian cancer is the most prevalent form of the disease, representing approximately 95% of all diagnosed cases. It consists of four subtypes: serous, endometrioid, mucinous, and clear cell. Serous epithelial ovarian cancer is further divided into high-grade serous carcinomas (HGSC) and low-grade serous carcinomas (LGSC). HGSC is the most common subtype, comprising about 70% of cases, while LGSC, endometrioid, mucinous, and clear cell subtypes account for roughly 5%, 10%, 3%, and 10%, respectively [6]. The standard of care for the treatment involves surgery, radiation therapy, and chemotherapy with platinum-based agents [7]. Despite advancements in ovarian cancer treatment, relapse remains a significant challenge for most patients with advanced disease, emphasizing the urgent need for more effective and durable therapeutic strategies. Targeted therapies, including PARP inhibitors and monoclonal antibodies like bevacizumab, have demonstrated clinical benefits and are currently employed as second-line treatments and maintenance therapies. However, their efficacy is heterogenous, and the emergence of resistance continues to limit long-term success [8, 9]. To address these limitations, tyrosine-kinase inhibitors (TKIs) such as nintedanib, sorafenib and sunitinib have gained attention as potential alternatives. Currently in phase II/III of clinical trials, they are showing promising results in improving survival rates for ovarian cancer patients [10]. Nevertheless, resistance mechanisms and incomplete tumor eradication remain major challenges, highlighting the need for complementary or alternative therapeutic approaches [10–12].

In this context, oncolytic viruses (OVs) – engineered to selectively target and destroy tumor cells while stimulating immune responses [13–20] – offer an opportunity as novel immunotherapeutic approach. Several OV candidates have progressed to clinical trials, with encouraging results [21]. Notably, ONCOS-102, a genetically modified oncolytic adenovirus expressing GM-CSF, has been evaluated in combination with checkpoint inhibitors and chemotherapy, demonstrating enhanced T-cell infiltration and durable anti-tumor responses in solid tumors, including ovarian cancer [21–24]. Despite these advances, OV monotherapy has shown modest clinical efficacy, likely due to the immunosuppressive tumor microenvironment (TME) and limited intratumoral viral replication [22–24]. Consequently, rational combinatorial approaches, integrating OVs with chemotherapy and anti-angiogenic therapies, have emerged as a promising avenue to enhance therapeutic efficacy in ovarian cancer [20, 25, 26].

The presented study explores the potential of the oncolytic adenovirus, Ad5/3-D24-ICOSL-CD40L, in combination with a triple-drug regimen based on cisplatin, paclitaxel, and nintedanib, as a novel treatment strategy for patients with ovarian cancer. The rationale for including nintedanib lies in its complementary mechanism of action with cisplatin and paclitaxel. While these two first-line agents target DNA damage and microtubule dynamics, nintedanib inhibits VEGFR, FGFR, and PDGFR signaling pathways shown to be overexpressed in our selected ovarian cancer models (A2780 and OVCAR-3), as confirmed by bioinformatic analyses (Human Protein Atlas) and supporting literature [27–30]. Thus, its inclusion allowed us to explore anti-angiogenic and signaling blockade effects in combination with cytotoxic and oncolytic therapies.

In general, traditional 2D cell culture inherently limits the advancement of cancer treatment by failing to replicate the complexity of the tumor microenvironment [31, 32]. This challenge becomes more pronounced when evaluating a combinatorial approach involving multiple agents with distinct mechanisms of action and administration routes. Therefore, we developed a dynamic three-dimensional (3D) based microfluidic platform using spheroids, derived from high-grade serous and endometrioid ovarian cancer cell lines, that better mimics the systemic administration and interactions of combinatorial therapies. While spheroids effectively replicate tumor architecture and cell-extracellular matrix (ECM) interactions, they lack key physiological dynamics such as fluid flow, immune cell infiltration, and drug perfusion, limiting their predictive power. Our microfluidic system enables dynamic perfusion, controlled nutrient and drug circulation, reproducing key functional aspects of microfluidic culture. This allows us to more closely mimic the in vivo tumor microenvironment and systemic drug exposure compared to static systems, main rationale for its use in our study. Specifically, this strategy integrates peripheral blood mononuclear cells (PBMCs) to simulate immune-tumor interactions and human umbilical vein endothelial cells (HUVECs) to incorporate angiogenic factors, thus providing a more accurate representation of the ovarian tumor microenvironment. Unlike static models where therapeutic agents remain stationary, this system allows for continuous perfusion, better resembling in vivo drug delivery and immune responses.

Our findings, obtained in 3D co-culture models on a dynamic microfluidic platform, demonstrate that oncolytic virus-mediated immune priming can enhance the efficacy of subsequent therapies leading to sustained tumor reduction and mitigating the rebound effect commonly observed after chemotherapy, suggesting the potential to extend therapeutic windows and reduce the likelihood of relapse. Overall, this study underscores the potential of combining oncolytic virotherapy with advanced drug delivery technologies to improve treatment outcomes in ovarian cancer, especially for patients with resistant or relapsed forms of the disease. This approach not only paves the way for evaluating more effective therapeutic strategies but also contributes to the ongoing quest for personalized treatment options in the fight against ovarian cancer.

## Materials and methods

### Cell lines, virus and antitumoral agents

The study was conducted on two human ovarian cancer cell lines obtained by the Biobank Graz. Human endometroid ovarian cancer A2780 cells were cultured in RPMI-1640 (Sigma-Aldrich) supplemented with 10% fetal bovine serum (FBS, Gibco Laboratories, USA), 1% penicillin/streptomycin (Gibco Laboratories, USA) and 1% L-glutamine (Gibco Laboratories, USA). Human ascitic high-grade serous ovarian cancer OVCAR-3 cells were cultured in RPMI-1640 (Sigma-Aldrich) supplemented with 20% of fetal bovine serum (FBS, Gibco Laboratories, USA), 1% penicillin/streptomycin (Gibco Laboratories, USA), 1% L-glutamine (Gibco Laboratories) and 0.01 mg/mL bovine insulin (Gibco Laboratories, USA). For the three-dimensional microfluidic platform studies, endothelial cells HUVECs pre-screened for vascular endothelial growth factor (VEGF) response and vWF (factor VIII antigen) were obtained by PromoCell (Heidelberg, Germany) and cultured in endothelial cell growth medium (PromoCell, Heidelberg, Germany).

The adenoviral vector used in this work underwent standard adenovirus preparation protocols for its generation and amplification, as previously described [20]. Briefly, three main modifications have been performed onto the Ad5 *wt* virus who served as backbone structure: (i) a 24-base pair deletion in the E1A conserved region; (ii) the insertion of the inducible costimulatory ligands ICOSL and CD40L under an exogenous promoter in the E3 region, (iii) Ad5/3 chimeric construct by alterations to the fiber knob. The Ad5/3-D24-ICOSL-CD40L and Ad5/3-D24-mCherry-ICOSL-CD40L (consisting of a 24-bp deletion in E1A Conserved Region 2 (CR2), a ICOSL-CD40L/mCherry-ICOSL-CD40L expression cassette inserted in the E3 region, and Ad5/3 hybrid fiber) have been produced, characterized and tested as described earlier [13, 33].

The alkylating agent cisplatin (CIS) was purchased from Santa Cruz Biotechnology (Dallas, TX, USA) and dissolved in milliQ water. The concentrations used for cisplatin in both two-dimensional (2D) and three-dimensional (3D) cell culture studies were 10 nM, 50 nM, 100 nM, 500 nM, 1 μM, 10 μM, 20 μM, 50 μM and 100 μM. The cytoskeletal drug and tubulin-targeting paclitaxel (PTX; Taxol, Selleck Chemicals, USA) and the multi-target tyrosine-kinase inhibitor nintedanib (NIN; Sigma-Aldrich, St. Louis, MO, USA) were both dissolved in DMSO. For 2D cell culture studies, paclitaxel was tested at concentrations of 1 nM, 10 nM, 100 nM, 500 nM, 1 μM, 2 μM, 5 μM and 10 μM; while nintedanib, was tested at 10 nM, 100 nM, 500 nM, 1 μM, 2 μM, 5 μM, 10 μM and 20 μM. In 3D cell culture studies paclitaxel was tested at 1 μM, 2 μM, 5 μM, 10 μM while nintedanib-cell culture medium solutions were tested at 2 μM, 5 μM, 7.5 μM, 10 μM. All drugs were prepared first as stock solutions and subsequently diluted in cell culture medium supplemented with 10% FBS to reach the desired concentration in each well.

### CAR, DSG-2 and CD46 expression in ovarian cancer cells

A2780 and OVCAR-3 cells, were seeded at a concentration of 5 × 10^5^ cells/well in 6-well plates and cultured in their respective culture media for 24 h, after which cells have been detached and stained with anti-CAR (Abcam, UK), anti-CD-46 (ThermoFisher, USA) and anti-DSG-2 (LSBio, USA) antibodies. Flow cytometric analysis was performed 30 min after incubation using BD FACSAria™ III (Becton Dickinson, USA) instrument. Further data analysis was performed through FlowJo software (V10).

### Screening of antitumoral agents’ cytotoxicity and LC50 evaluation

A2780 and OVCAR-3 cells were seeded at a concentration of 1 × 10^4^ cells/well in a 96 well plate with flat bottom and maintained under standard cell culture growth conditions overnight. A range of antitumoral agents above described was tested on both human ovarian cancer cell lines. For the oncolytic adenovirus Ad5/3-D24-ICOSL-CD40L the tested concentrations were: 0.1, 1, 10, 100, 1000 VP/cell. Cell viability was carried out 72 h after treatment, using CellTiter 96 Aqueous One Solution Cell Proliferation Assay (MTS) in accordance with manufacturer’s instruction (Promega, Madison, WI, USA). The absorbance was measured at 490 nm using a 96-well plate spectrophotometer (Victor Nivo™, Perkin Elmer). The experiment was performed in triplicate. The LC50 (lethal concentration 50) value for each treatment was then calculated interpolating the average values with a logarithmic function setting y = 50, finding the corresponding value on the x-axis, representing the LC50 range.

### Cell viability: MTS cytotoxicity assay

A2780 and OVCAR-3 cells were seeded at a concentration of 1 × 10^4^ cells/well in a 96 well plate with flat bottom and maintained under standard cell culture growth conditions. After incubation overnight, cells were treated as presented in Table 1. Cell viability assay was assessed 72 h after treatment using CellTiter 96 Aqueous One Solution Cell Proliferation Assay (MTS) in accordance with manufacturer’s instruction (Promega, Madison, WI, USA). The absorbance was measured at 490 nm using a 96-well plate spectrophotometer (Victor Nivo™, Perkin Elmer). The experiment was performed in triplicate.

**Table 1:** Treatment schedule for the assessment of tested anti-cancer agents in vitro

Treatment conditions	Day 1 (24 h after seeding)	Day 2 (48 h after seeding)
Ad5/3-D24-ICOSL-CD40L (OAdV)	100 VP/cell	Treatment removal
Paclitaxel (PTX)	500 nM	
Cisplatin (CIS)	1 μM	
Nintedanib (NIN)	1 μM	
CIS + PTX + NIN	CIS 1 μM + PTX 500 nM + NIN 1 μM	
OAdV + CIS + PTX + NIN	100 VP/cell + CIS 1 μM + PTX 500 nM + NIN 1 μM	
Priming OAdV + CIS + PTX + NIN	100 VP/cell	CIS 1 μM + PTX 500 nM + NIN 1 μM

### Immunogenic cell death (ICD) markers evaluation

*ATP release*. Ovarian cancer cells were seeded on a 96-well plate at a concentration of 1 × 10^4^ cell/well with the corresponding growth medium. On the following day, cells have been treated as outlined in Table 1. 96 h post treatment, cells have been analyzed with ATP determination kit CellTiter-Glo® Luminescent Cell Viability Assay (Promega), according to manufacturer’s protocol. The luminescence was assessed with the spectrophotometer (Victor Nivo™, Perkin Elmer).

*CRT exposure*. Ovarian cancer cells were seeded onto 24-well plates at a density of 5 × 10^4^ cells/well and maintained under standard growth conditions. On the following day, cells were treated as described in Table 1*.* Then, 48 h after treatment, cells were harvested and stained with 1:1000 diluted Alexa-Fluor 488 rabbit polyclonal anti-calreticulin antibody (Abcam, Cambridge, UK) (concentration of 1 μg/mL) or Alexa Fluor Plus 488 goat anti-mouse at the concentration 1–10 μg/mL (ThermoFisher, Scientific, A32723, Waltham, MA, USA) for 30 min and analyzed by flow cytometry analysis using BD FACSAria™ III instrument (New Jersey, USA). The experiments were independently conducted three times, with three replicates for each treatment.

### Confocal microscopy studies

Ovarian cancer cells were seeded on 24-chamber glass slides (Sigma–Aldrich, St. Louis, MO, USA) in a 24 well plate at a concentration of 1 × 10^5^ cells/well in the appropriated cell culture medium. After 24 h cells were treated as follows: *(i)* culture media, *(ii)* paclitaxel (500 nM), *(iii)* cisplatin (1 μM), *(iv)* nintedanib (1 μM), *(v)* Ad5/3-D24-mCherry-ICOSL-CD40L (100 VP/cell), *(vi)* Ad5/3-D24-ICOSL-CD40L + CIS 1 μM + PTX 500 nM + NIN 1 μM and *(vii)* priming Ad5/3-D24-mCherry-ICOSL-CD40L + CIS 1 μM + PTX 500 nM + NIN 1 μM. After incubation with the different treatments for 24, 48, 72 and 96 h, cells were washed three times with PBS (Aurogene, Rome, Italy) and then fixed with 4% (w/v) paraformaldehyde in PBS for 15 min. Nuclei were stained with Hoechst (Thermo Scientific, Waltham, Massachusetts, USA) diluted 1:1000 in PBS and incubated for 10 min. Cells were washed three times with PBS between these steps. Cover glasses were removed from plates and mounted in microscope slides (Menzel-Glaser, Thermo Scientific, Waltham, Massachusetts, USA) using Prolong™ Glass Antifade mountant (InVitrogen, Waltham, Massachusetts, USA). A confocal analysis was performed by Zeiss LSM800 (Zeiss, Oberkochen, Germany) equipped with 63 × oil objective. Lasers at 385/30 nm and 631/33 nm were used to detect the fluorescence of Hoechst and mCherry, respectively. Image analyses were carried out using ImageJ.

### Establishment of 3D cell culture

In order to allow spheroids formation, ovarian cancer cell lines were seeded at different density (100, 200, 400, 600, 800, 1000, 2000, 3000, 5000, 7500, 10,000 cells/well) in 96 well plate with flat bottom, previously coated with 50 μL of an 1.5% agarose solution (Thermo Fisher Scientific, Waltham, Massachusetts, USA). After seeding, plates were centrifuged at 1000 rpm for 5 min using Micro Star 30R (VWR, USA) centrifuge. Then, spheroid’s morphology and growth were monitored using Axiovert 40 CFL microscope (Zeiss, Oberkochen, Germany) for 9 days both for A2780 and OVCAR-3 cell lines. Spheroid areas were registered every three days using imaging software (AxioVision software by Zeiss, Oberkochen, Germany).

### 3D cell culture spheroids: antitumoral agents screening

A2780 and OVCAR-3 cells were seeded on a 96 well plate previously coated with 50 μL of an 1.5% agarose solution at a concentration of 400 and 1000 cells/well, respectively. Three days after spheroids formation, antitumoral agents were administered every three days (four times in total); then, the cell culture medium was changed once a week up to day 33. Screening range was above-mentioned in Paragraph 2.1 of Materials and methods. Spheroid areas were registered every three days using imaging software (AxioVision software by Zeiss, Oberkochen, Germany).

### 3D cell culture spheroids: combination treatments

A2780 and OVCAR-3 cells were seeded on a 96 well plate previously coated with 50 μL of an 1.5% agarose solution at a concentration of 400 and 1000 cells/well, respectively. Three days after spheroids formation, antitumoral agents were administered every three days (four times in total); then, the cell culture medium was changed once a week up to day 33. Treatments schedules are outlined in Table 2. Spheroid areas were registered every three days using imaging software (AxioVision software by Zeiss, Oberkochen, Germany).

**Table 2:** Treatment schedule for 3D cell culture ovarian cancer models

Treatment conditions	Day 4	Day 6	Day 8	Day 10	Day 12 and every 2 days thereafter
Ad5/3-D24-ICOSL-CD40L (OAdV)	10^8^ VP/cell	10^8^ VP/cell	10^8^ VP/cell	10^8^ VP/cell	Treatment removal and medium change once a week Follow up until day 33
Paclitaxel (PTX)	2 μM	2 μM	2 μM	2 μM	
Cisplatin (CIS)	500 nM	500 nM	500 nM	500 nM	
Nintedanib (NIN)	5 μM	5 μM	5 μM	5 μM	
CIS + PTX + NIN	500 nM + 2 μM + 5 μM	500 nM + 2 μM + 5 μM	500 nM + 2 μM + 5 μM	500 nM + 2 μM + 5 μM	
Co-adm: OAdV + CIS + PTX + NIN	10^8^ VP/cell + 500 nM + 2 μM + 5 μM	10^8^ VP/cell + 500 nM + 2 μM + 5 μM	10^8^ VP/cell + 500 nM + 2 μM + 5 μM	10^8^ VP/cell + 500 nM + 2 μM + 5 μM	
Priming: OAdV + CIS + PTX + NIN	10^8^ VP/cell	500 nM + 2 μM + 5 μM	10^8^ VP/cell	500 nM + 2 μM + 5 μM	Repeat the treatment modality every 2 days up to day 18 and then follow up until day 33

### Establishment of 3D cell culture spheroids using hanging drop method

To generate spheroid for the enforced 3D model using the microfluidic device, the hanging drop method was employed, facilitating cell aggregation and spheroid formation under gravity-driven conditions. Briefly, cells were harvested and counted in a concentration of 400, 600, 800 and 1000 cells/drop. Using a sterile pipette, a drop was formed containing the desired number of cells in a volume of 10 μL and 20 μL. The drop was carefully placed on the inner part of a Petri dish lid (TC Dish 60, Standard, Sarstedt, Nümbrecht, Germany). The lid was then inverted and positioned on the bottom part of the Petri dish, where cell culture medium was previously placed to maintain humidity and prevent drop evaporation. The Petri dish was closed and left in the incubator for about 6 days, then hanging drops spheroids were gently transferred in the upper chamber of the microfluidic bioreactor LiveBox2 (IVTech Srl., Massarosa, LU, Italy).

### Establishment of 3D coculture of ovarian cancer cells with peripheral blood mononuclear cells (PBMCs) and human umbilical vein endothelial cells (HUVECs)

Peripheral blood mononuclear cells (PBMCs) were purchased from StemCell Technologies (Vancouver, British Columbia, Canada) and stored in liquid nitrogen until use. Human umbilical vein endothelial cells, prescreened to demonstrate stimulation-dependent angiogenesis were purchased from PromoCell (Heidelberg, Germany) and stored in liquid nitrogen until use. A2780 and OVCAR-3 cells were stained with carboxyfluorescein succinimidyl ester (CFSE, Sigma-Aldrich, Saint Louis, MO, USA) according to the manufacturer’s instructions, while the CellTracker Orange CMTMR (C2927, Invitrogen, Waltham, Massachusetts, USA) was used to stain PBMCs, following the manufacturer’s instructions. Blue CMAC (Thermo Scientific, Waltham, Massachusetts, USA) was used to stain HUVECs. A2780 and OVCAR-3 cell lines, previously stained with cell trace CFSE, were seeded at a density of 400 cells/well and 1000 cells/well in 96-well plates with a flat bottom, previously coated with 50 μL of a 1.5% agarose solution (Thermo Scientific, Waltham, Massachusetts, USA). After seeding, the plates were centrifuged at 1000 rpm for 5 min to allow spheroid formation. On day 6, the coculture system was assembled by introducing PBMCs and HUVECs, pre-stained with CellTracker Orange and Blue CMAC, respectively. The cells were added to the spheroids at a 1:5 PBMCs-to-spheroid ratio to promote immune cell infiltration, while HUVECs were incorporated at a 1:5 ratio relative to PBMCs to enhance tumor vascularization and drug delivery. After 24 h, fluorescence images were acquired using a 10 × objective on a Nikon Ts fluorescence microscope.

### Establishment of a microfluidic 3D in vitro model for ovarian cancer

**Fig. 1:**
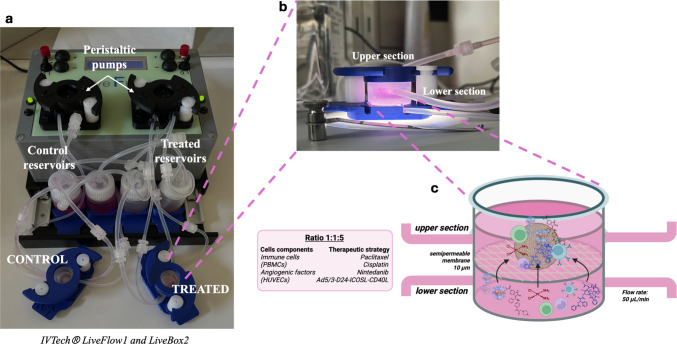
Microfluidic IVTech system setting. **a** Overview of the IVTech® LiveFlow1 and LiveBox2 system, which includes peristaltic pumps, control and treated reservoirs, and fluidic connections for maintaining flow conditions. **b** Close-up of the LiveBox2 chamber, showing the upper and lower sections where immune cells and treatments interact. **c** Schematic representation of the system: the upper section holds the tumoral spheroid, separated by a semipermeable membrane (10 µm) from the lower section, which receives fresh medium, treatments, immune cells (PBMCs) at a 1:5 ratio and angiogenic factors (HUVECs) at a 1:5 ratio, and at a controlled flow rate of 50 µL/min

A microfluidic system (IVTech Srl., Massarosa, LU, Italy) was utilized for this study. The device comprises two peristaltic pumps (LiveFlow®, IVTech Srl., Massarosa, LU, Italy) that generate the flow, connected to two modular flow bioreactors named LiveBox2 (LB2), where spheroids are cultured. The pumps and LB2 bioreactors are interconnected through silicon tubing and sealed to them by manufacturer’s, with plastic bottles serving as reservoirs for medium or treatment discard. The LB2 bioreactor is a dual flow-system composed of two chambers, an upper one (*apical*, wet total volume of 1.5 mL, including an inlet and an outlet tube manually connected to the peristaltic pumps by tubing) and a lower (*basal*, wet total volume of 1 mL, including an inlet and an outlet tube manually connected to the peristaltic pumps by tubing) chamber, designed to replicate physiological barriers an in vitro model. The two chambers are divided by a membrane holder with the aim of supporting a semi-permeable polyethylene terephthalate (PET) membrane, with a porosity of 10 µm (Fig. 1). The two parts of LB2 modular flow bioreactors can be manually separated to insert the semipermeable membrane, allowing the placement of the spheroid on top of it. The upper section is equipped with a blue cap (Fig. 1a-b) that can be manually operated to allow the position the spheroid within the system. Once placed, the system is sealed manually by mechanically tightening the upper section cap. The entire setup can be placed inside an incubator to maintain optimal culture conditions in terms of CO_2_ and humidity (5% CO_2_ and 37 °C).

### Microfluidic-based 3D coculture model

Ovarian cancer-derived spheroids generated by hanging drop method (see Paragraph 2.9 of Materials and Methods) were gently transferred in the upper chamber of the microfluidic bioreactor LiveBox2 (IVTech Srl., Massarosa, LU, Italy) placed on the semipermeable membrane. Then, to set up a coculture 3D ovarian cancer microfluidic based model, PBMCs from healthy donors, HLA matching with the selected ovarian cancer cells (HLA A2780: A*26:03 and OVCAR-3: A*02:01) and HUVECs, prescreened to demonstrate stimulation-dependent angiogenesis, were diluted in the appropriate cell culture medium in ratio 1:5 and introduced into the lower chamber at a flow rate of 50 µL/min (Fig. 1). In this way, immune cells and angiogenic factors are expected to flow in the upper chamber and infiltrate in the tumor cell spheroid. The spheroids were subjected to two distinct treatment protocols: the priming protocol and the co-administration protocol, while within untreated control spheroids fresh medium was flowed. In the lower chamber, treatment solutions were administered according to Table 3. Both control and treated spheroids were subsequently analyzed to assess the effects of the different treatment approaches. The duration of each treatment was determined based on the European Public Assessment Report (EPAR) guidelines for cisplatin, nintedanib and paclitaxel [34–36]. The virus and the drugs were dissolved, from stock solutions, in medium culture to obtain the proper concentrations. Spheroids were monitored for up to 31 days using a stereomicroscope (Optika, BG, Italy) and the area was registered using ImageJ (Fiji, NIH, USA). Spheroids were monitored for up to 31 days using a stereomicroscope (Optika, BG, Italy) and the area was registered using ImageJ (Fiji, NIH, USA. The experiment was performed in duplicate.

**Table 3:** Treatment schedule for the microfluidic-based 3D in vitro model

Treatment condition	Intervention (every 72 hours)			Endpoint
Control	Fresh medium			Repeat every 72 hours until day 19 and then follow up
Co-administration	OAdV + PTX + CIS + NIN (48 hours)		Fresh medium (24h)	
Priming	OAdV (48 hours)	Fresh medium (24h)	PTX + CIS + NIN (10 hours)	

### Statistical analysis

Data were reported as mean ± SEM or as indicated. Statistical analysis was performed with GraphPad Prism software version 10.2 (La Jolla, CA, USA). One-way Anova, Two-way Anova tests were used to compare two or more groups.

## Results and discussion

### Comparative evaluation of antitumoral agents as monotherapies in ovarian cancer models

Preclinical studies have provided critical insights into the potential of oncolytic viruses as promising therapies for ovarian cancer [37]. However, their efficacy is significantly influenced by the tumor microenvironment (TME), which is often highly immunosuppressive, limiting viral replication and immune activation [38]. While combination therapies integrating OVs with chemotherapy have demonstrated anticancer efficacy [14, 20, 39], their evaluation has largely relied on traditional two-dimensional (2D) monolayer cultures or static three-dimensional (3D) spheroid models. These, fail to recapitulate the physiological complexity of an in vivo tumor, particularly in terms of systemic drug perfusion, immune cell infiltration, and tumor-immune interactions [40].

To address these limitations, this study aimed to develop and validate an advanced 3D microfluidic platform capable of mimicking in vivo-like tumor conditions and assessing the efficacy of Ad5/3-D24-ICOSL-CD40L in combination with cisplatin, paclitaxel and nintedanib. This dynamic tumor-on-a-chip system integrates ovarian cancer derived spheroids, peripheral blood mononuclear cells (PBMCs), and angiogenic factors from HUVECs, allowing for real-time monitoring of drug response, immune interactions, and viral efficacy under continuous flow conditions. Utilizing this platform, we aimed to: (i) assess the therapeutic potential of the oncolytic virus in combination with chemotherapeutic and targeted agents under dynamic perfusion conditions that more accurately mimic in vivo drug exposure and tumor perfusion; (ii) compare treatment strategies by evaluating two administration protocols—priming versus co-administration—to identify the regimen that maximizes tumor cytotoxicity; and (iii) characterize tumor-immune interactions with a focus on immunogenic cell death (ICD) markers, including extracellular ATP release and calreticulin surface exposure.

Overall, the immunosuppressive microenvironment of ovarian tumors can limit the effectiveness of these treatments. We have previously demonstrated that Ad5/3-D24-ICOSL-CD40L, which encodes two co-stimulatory molecules—ICOSL and CD40L—can significantly influence anticancer immune responses [14–16, 20]. This capability enables a shift from tumor immunosuppression to immunomodulation, thereby enhancing the overall immune response [41, 42]. A key determinant of OV efficacy is the expression of adenoviral entry receptors, which indicate viral internalization, replication and oncolysis [37]. To assess the feasibility of Ad5/3-D24-ICOSL-CD40L for ovarian cancer treatment, we first characterized the expression of three primary adenoviral receptors in A2780 (endometrioid) and OVCAR-3 (high-grade serous) ovarian cancer cell lines: coxsackievirus and adenovirus receptor (CAR), desmoglein-2 (DSG-2), and CD46, which facilitate viral entry [15, 37, 43]. This investigation is based on the premise that consistent and significant expression of these receptors facilitates greater internalization of viral particles, leading to enhanced infectivity [37]. Both the ovarian cancer cells expressed high levels of positive cells for adenoviral receptors (CAR: 79%, DSG-2: 94%, CD46: 96% for A2780 and CAR: 94.1%, DSG-2: 99%, CD46: 98% for OVCAR-3, respectively) (Supplementary Fig. 1). Mean fluorescence intensity (MFI) in both A2780 and OVCAR-3 selected cell lines, DSG-2 resulted to be overexpressed (A2780: 751 and OVCAR-3: 7471.5, respectively) compared to CAR (A2780: 382.5 and OVCAR-3: 1432, respectively) and CD46 (A2780: 472.5 and OVCAR-3: 1575, respectively) (Supplementary Fig. 1). The flow cytometric analysis confirmed high receptor expression in both tested cell lines, with DSG-2 being the most overexpressed receptor. Collectively, the obtained results are in agreement with previous findings reporting that oncolytic adenovirus internalization mainly occurs via interaction with DSG2 receptors [37, 43]. Taken together, these data suggest that both tested ovarian cancer subtypes are highly susceptible to Ad5/3-D24-ICOSL-CD40L infection, highlighting its potential for broad applicability across different ovarian cancer phenotypes.

To assess the feasibility of a combination therapy based on Ad5/3-D24-ICOSL-CD40L, cisplatin, nintedanib and paclitaxel for the treatment of ovarian cancer, we first needed to conduct a proper screening of each drug in monotherapy before evaluating the potential anti-cancer efficacy of the combination therapy in two-dimensional (2D) cell culture models (Supplementary Fig. 2). The aim of the experiment was to determine the lethal concentration (LC50) for each antitumoral agent, in order to select the proper dosages for further establishing the proposed combinatorial approach. Treatment dosages for screening were determined both from literature and previous research by the group [13, 20], [44–49]. The results demonstrated a dose-dependent decrease in cell viability across both ovarian cancer cell lines, confirming the cytotoxic efficacy of the tested agents (Supplementary Fig. 2a-h). This trend was further validated through LC50 (lethal concentration 50) analysis (Supplementary Fig. 2i), which provided quantitative insights into the potency of each treatment. Notably, while A2780 and OVCAR-3 cells exhibited similar sensitivity to most agents, OVCAR-3 displayed greater resistance to cisplatin (Supplementary Fig. 2a-h), suggesting potential intrinsic differences in chemoresistance mechanisms between the two subtypes.

The LC50 values were determined using logarithmic interpolation, with y set to 50, allowing precise identification of the concentration range required for 50% cell viability reduction. Based on these findings, and to maximize the potential for combinatorial effects in multimodal therapy, the following concentrations were selected for subsequent studies: paclitaxel (500 nM), cisplatin (1 µM), nintedanib (1 µM), and Ad5/3-D24-ICOSL-CD40L (100 VP/cell). These doses were chosen to ensure sufficient cytotoxic activity while maintaining a physiologically relevant concentration for combinatorial approaches.

### Evaluation of cell cytotoxicity and transduction efficiency in 2D ovarian cancer cellular models

**Fig. 2:**
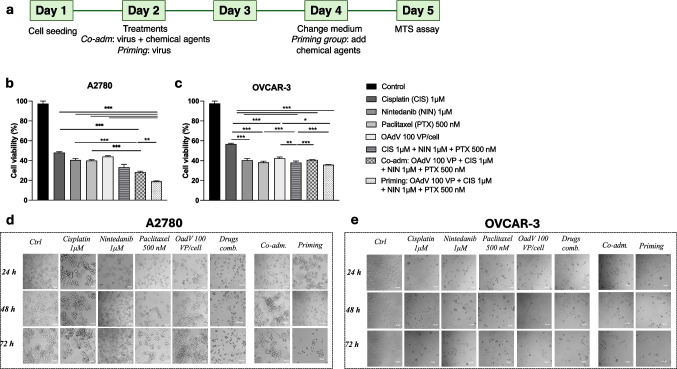
Evaluation of combination therapy through MTS cytotoxic assay. **a-c** Different combinations of Ad5/3-D24-ICOS-CD40L (100 VP/cell), cisplatin (1 μM), nintedanib (1 μM), paclitaxel (500 nM) were tested on A2780 and OVCAR-3. After treatment, percentage of viable cells was determined using CellTiter 96 Aqueous One Solution Cell Proliferation Assay (MTS) and measuring the absorbance at 490 nm with a 96-well plate spectrophotometer Viktor Nivo tm. Statistical analysis was performed with one-way ANOVA (*P ≤ 0.01, **P ≤ 0.001, ***P ≤ 0.0001). **d-e** Representative images of the treatment conditions obtained using an optic microscope using a 10 × objective

Monotherapy rarely proves effective in cancer treatment [50]. Research suggests that combining various therapies can significantly enhance treatment outcomes [13, 15, 51, 52]. In light of this, we aimed to assess the anticancer efficacy of Ad5/3-D24-ICOSL-CD40L when used alongside a triple-drug regimen consisting of paclitaxel, cisplatin and nintedanib. To this end, MTS cell viability assays were conducted on ovarian cancer cells (Fig. 2b-c). After identifying the appropriate dosages for each antitumor agent (Supplementary Fig. 2), we explored two distinct administration strategies. The first involved the simultaneous *co-administration* of the triple-drug therapy and the oncolytic adenovirus. The second approach, named *priming*, considered the viral absorption time necessary for attachment to the cell surface and subsequent internalization. To implement this, the initial step was to administer the virus alone as a priming agent, followed by the introduction of the triple-drug treatments 48 h later. This timing allowed the virus to exert its anticancer effects before the drugs were introduced. The treatment scheme for ovarian cancer cells is presented in Table 1. The results indicate decreased cell viability using the priming treatment schedule compared to the co-administration protocol in both cell lines (A2780, co-administration: 28.3% and priming: 18.8%; OVCAR-3, co-administration: 35.2% and priming: 31.5%) (Fig. 2b-c). These findings agree with previous observations that indicated the priming protocol significantly induced higher tumor cell death compared to the co-administration scheme [20].

**Fig. 3:**
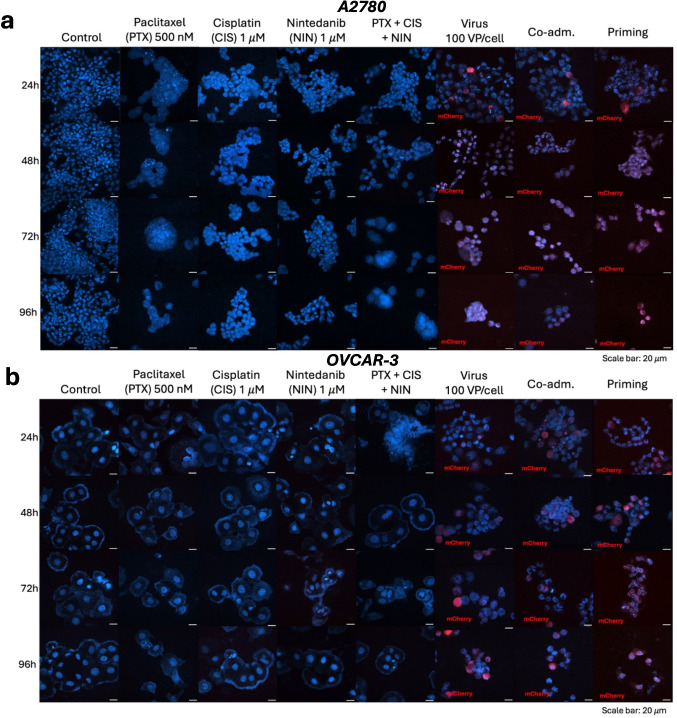
Transduction efficiency assessed by confocal microscopy on A2780 (**a**) and OVCAR-3 (**b**) cell lines. Paclitaxel, cisplatin nintedanib and AdV5/3-D24-ICOSL-CD40-mCherry were administered alone or in combination. After incubation for 24, 48, 72 and 96 h, the cells were washed three times with PBS (Aurogene) and fixed with 4% (w/v) paraformaldehyde in PBS for 15 min. The nuclei were stained with Hoechst (Thermo Scientific) diluted 1:1000 in PBS and incubated for 10 min. Confocal analyses were conducted using a Zeiss LSM800 microscope with 63 × objective

Confocal analysis was performed to assess the effect of Ad5/3-D24-ICOSL-CD40L (expressing mCherry) and its combination with the triple-drug regimen on ovarian cancer cells, by the assessment of the transfection through fluorescent protein visualization (Fig. 3). The progress of the viral infection could be monitored by detecting the emission signal of the protein fluorophore mCherry (excitation λ: 540–590 nm; emission λ: 550–650) which is expressed by the virus. The more intense the emission of this signal, the greater the concentration of the fluorophore, and consequently the presence of the actively replicating oncolytic virus inside tumour cells. Confocal microscopy revealed viral internalization after 48 h post-infection in both tested cell lines, occurring in the absence and presence of the antitumoral agents, suggesting viral uptake regardless of treatment conditions. Notably, both co-administration and priming protocols allowed the oncolytic vector to replicate, demonstrating that the presence of chemical agents doesn’t hinder its activity.

The priming protocol demonstrated superior viral uptake and cytotoxic efficacy, as evidenced by the higher fluorescence intensity of mCherry expression (Fig. 3a-b). This suggests that allowing a pre-exposure window for Ad5/3-D24-ICOSL-CD40L before targeted and chemotherapy administration enhances viral internalization and replication, likely due to improved viral-host interactions and reduced competition with cytotoxic agents for cellular entry. In contrast, co-administration may have led to suboptimal viral infection, as the simultaneous presence of chemotherapy could have compromised cell viability before the virus had sufficient time to establish replication.

Further supporting these observations, distinct morphological changes were evident upon drug treatments, aligning with the known mechanisms of action of each agent. Paclitaxel, a microtubule depolymerization inhibitor, induces mitotic arrest at metaphase, leading to prolonged spindle checkpoint activation and subsequent apoptosis. This effect was particularly pronounced in A2780 cells, where characteristic mitotic figures were observed, consistent with prior findings reported by Kuryk et al. [20]. Cisplatin, a DNA crosslinking agent, disrupted nuclear integrity, leading to chromatin condensation and fragmentation, indicative of apoptotic cell death [45]. Nintedanib, a multi-target tyrosine kinase inhibitor, effectively suppressed cell proliferation by inhibiting angiogenic and pro-survival pathways, leading to widespread tumor cell shrinkage and detachment [46]. The combined effects of these agents resulted in a significant reduction in viable cell numbers, with clear morphological indicators of cytotoxicity, including cell shrinkage, rounding, and detachment from the culture surface (Fig. 3a-b).

Comparing the two administration strategies, the priming protocol consistently exhibited superior cytotoxicity, reinforcing the hypothesis that sequential OV priming enhances treatment efficacy. This may be attributed to the time-dependent increase in viral replication, allowing the virus to exert its oncolytic effects before chemotherapy-induced stress further amplifies tumor cell destruction. These findings provide compelling preclinical evidence that a priming-based strategy could optimize oncolytic virotherapy-chemotherapy combinations, potentially improving clinical outcomes in ovarian cancer treatment.

### Immunogenic cell death assessment

**Fig. 4:**
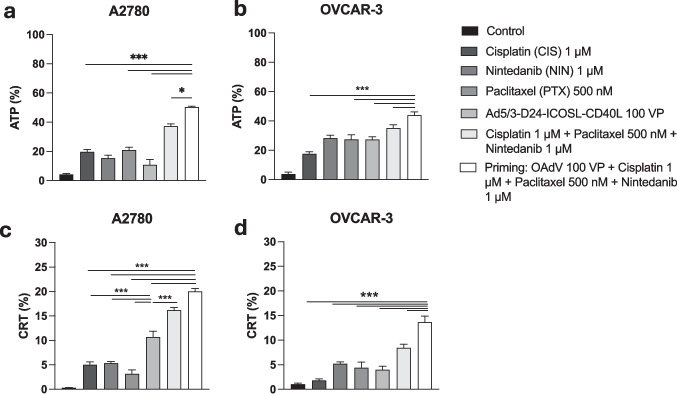
Immunogenic cell death assessment on ovarian cancer cells. **a-b** ATP levels were detected 72 h after infection with combinations of: Ad5/3-D24-ICOS-CD40L (100 VP/cell), cisplatin (1 μM), nintedanib (1 μM), paclitaxel (500 nM) on A2780 and OVCAR-3. Data are expressed as the percentage of extracellular ATP, assessed ATP determination kit CellTiter-Glo® Luminescent Cell Viability Assay (Promega), the luminescence was then assessed with Viktor Nivo.™. **c-d** Calreticulin (CRT) levels were detected 48 h after infection with combinations of Ad5/3-D24-ICOS-CD40L (100 VP/cell), cisplatin (1 μM), nintedanib (1 μM), paclitaxel (500 nM) on A2780 and OVCAR-3. Data are expressed as the percentage of positive cells for CRT, measured by flow cytometry (BD FACSAria III, Becton Dickinson, USA) followed by specific antibody staining. Statistical analysis was performed with one-way ANOVA (*P ≤ 0.01, ***P ≤ 0.0001)

In order to assess whether the tested treatments induced immunogenic cell death (ICD), we measured two established markers in ovarian cancer cells: calreticulin (CRT) surface exposure and extracellular ATP release, following the treatment protocol outlined in Table 1. The combinatorial therapy, particularly under the priming regimen, significantly increased ICD marker expression compared to monotherapies or the triple-drug combination without the virus. Specifically, CRT exposure reached 20% in A2780 and 21% in OVCAR-3, while ATP release levels were 50% and 43%, respectively (Fig. 4).

Among the chemotherapeutics, calreticulin exposure was minimal—especially with cisplatin—aligning with previous evidence that it does not induce CRT translocation, unlike oxaliplatin [47]. In contrast, ATP release was more pronounced across both the individual antitumoral agents and the oncolytic virus. Notably, Ad5/3-D24-ICOSL-CD40L alone triggered substantial ATP release, consistent with prior findings from our group in mesothelioma and breast cancer models [13, 20].

These results support the notion that the priming strategy enhances ICD induction, likely by allowing sufficient viral replication and immunostimulation prior to the cytotoxic impact of chemotherapy. The observed increases in CRT exposure and ATP release align with the enhanced antitumor efficacy seen in spheroid size reduction (Fig. 2–3), reinforcing the potential of OV priming to precondition the tumor microenvironment and promote effective immuno-chemovirotherapy.

### Three-dimensional (3D) ovarian cancer models

Three-dimensional (3D) models serve as a bridge between traditional two-dimensional (2D) cell cultures and in vivo model [53] by more accurately replicating the complexity and composition of the tumor microenvironment (TME). Even though two-dimensional (2D) cell culture models constitute the most implemented method to test the efficacy of a therapeutic approach, they are not able to accurately replicate tumor complexity and microenvironment [54]. Consequently, to further explore the efficacy of proposed anticancer treatments for ovarian cancer, human 3D spheroids were developed to emulate a hypothetical in vivo treatment scenario. This involved multiple administrations of drug agents and long-term monitoring, which included assessing spheroid integrity and growth kinetics by measuring both the areas (μm^2^).

**Fig. 5:**
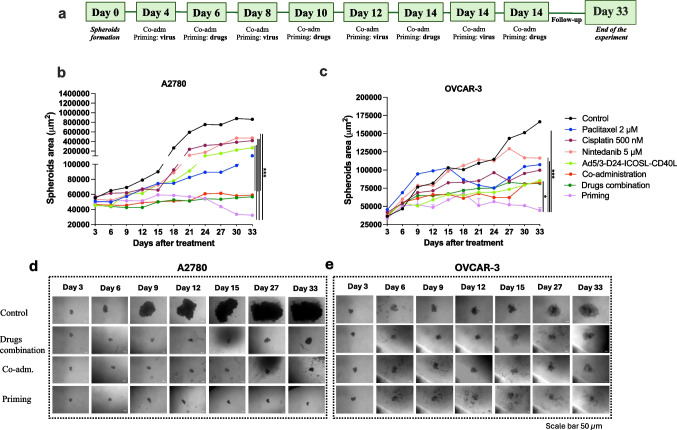
Assessment of anticancer efficacy of combination therapy on human ovarian cancer spheroids. **a** A2780 and OVCAR-3 cells were seeded at a density of 400 cells/well and 1000 cells/well, respectively in a 96-well plate with 1.5% agarose and centrifuged at 1000 rpm for 5 min to form spheroids. On day 4, spheroids were treated according to Table 2. Spheroids (n = 4/group) were examined microscopically every three days until day 33. Statistical analysis was performed using one-way ANOVA (***P ≤ 0.0001). **b-c** Spheroid size was measured on two dimensions every three days using Zeiss AxioVision software. **d-e** Representative optic microscope pictures of the treated spheroids throughout the experiments

A first screening to determine the optimal number of cells needed to create ovarian cancer-based spheroids of the desired size and morphology was performed (A2780: 400 cells/well; OVCAR-3 1000 cells/well) (Supplementary Fig. 3). Then, a dose screening for the antitumoral agents was conducted (Supplementary Fig. 4) based on the two-dimensional LC50 values (Supplementary Fig. 2i) as a reference. Given the increased complexity of 3D systems, the screening was focused on doses higher than the LC50 values. Therefore, ovarian cancer spheroids were established, then, it was hypothesized that the optimal time to initiate various treatments would be around day 4 post-spheroid formation, as all spheroids would be in the linear growth phase. Therefore, spheroids received a multiple-administration treatment of the drug agents with a long-term follow-up including the measurement of spheroid growth kinetics by registering the area (μm^2^). Treatment schedule of the 3D ovarian cancer models is outlined in Table 2. The combination dosages were selected for the antitumoral agents (cisplatin: 1 µM; paclitaxel: 5 µM; nintedanib:10 µM) while an intermediate viral titer of 10^8^ VP/mL was chosen for the oncolytic adenovirus. This choice aligns with the intent to avoid overshadowing the antitumoral agents, compromising the rationale behind the combinatorial approach. The results obtained confirm the improved anticancer efficacy of the combination strategy compared to monotherapies in both cell lines (Fig. 5b-c). Monotherapies demonstrated a limited efficacy in controlling the growth of spheroids, whereas combinations (both co-administration and priming) showed significant reductions in area measurements. (Fig. 5b-c). Notably, across all the combinations, the effect of the co-administration was comparable to that of the drug combination without the virus. This result supports the hypothesis that, in the co-administration protocol, the virus doesn’t have enough time to exert its cytotoxic activity effectively (Fig. 5b-c). The selected dosage (Fig. 5) was the most successful in reducing spheroid areas (Fig. 5d-e), where a “scissor” trend has been detected 27 days after treatment where a continued reduction is visible by the priming protocol (A2780 area day 33: 28,000 µm^2^; OVCAR-3: 44,410 µm^2^) and a rebound effect of both co-administration (A2780 area day 33: 64,500 µm^2^; OVCAR-3: 82,800 µm^2^) and drug combinations only (Fig. 5). Collectively, the priming protocol may not only serve to enhance the antitumoral activity but also to extend the duration of treatments, potentially preventing rebound effects, a common side effect related to chemotherapy. Although nintedanib is not currently recommended as part of standard or neoadjuvant therapy for ovarian cancer, our findings indicate that when combined with chemotherapeutic and oncolytic agents, it can exert complementary antitumor effects, suggesting its potential to contribute to new therapeutic strategies for this challenging malignancy.

### Microfluidic-based 3D co-culture model for evaluating combination therapies in ovarian cancer

The limitations of traditional 2D monolayer cultures have prompted the development of 3D tumor models, which more accurately recapitulate tumor architecture, drug penetration, and cell–cell interactions. In this study, a commercially available microfluidic platform (IVTech Srl., Massarosa, LU, Italy) was employed and customized by integrating ovarian cancer spheroids, PBMCs, and HUVECs to reproduce a dynamic tumor–immune–endothelial microenvironment for the evaluation of multimodal therapeutic regimens. We employed a microfluidic 3D culture model to better simulate the ovarian tumor microenvironment (TME) and to evaluate the therapeutic efficacy of Ad5/3-D24-ICOSL-CD40L in combination with a triple-drug regimen in a physiologically relevant setting [50]. Unlike static 3D spheroid models, which lack fluid dynamics and temporal resolution, microfluidic platforms enable continuous perfusion of nutrients, therapeutic agents, and immune cells. This dynamic environment enhances the fidelity of tumor-immune interactions and significantly improves the predictive accuracy of preclinical drug screening [20, 49].

The microfluidic model developed in this study integrates relevant immune components, particularly PBMCs and angiogenic factors from HUVECs, to assess efficacy of investigated therapies in a controlled microenvironment. By incorporating key physiological parameters, such as blood flow dynamics and immune-tumor crosstalk, this system allows a more precise evaluation of the immunomodulatory potential of Ad5/3-D24-ICOSL-CD40L, an engineered OV [13–20]. Indeed, the immunomodulatory effect in our system is primarily attributed to the oncolytic adenoviral vector Ad5/3-D24-ICOSL-CD40L, which encodes two immune co-stimulatory ligands, ICOSL and CD40L. These molecules interact with their respective receptors on PBMCs, promoting T-cell activation, antigen presentation, and cytokine release, thereby initiating an immune-stimulatory cascade within the co-culture. We have previously demonstrated this effect in another cancer model, where the same adenoviral vector induced measurable immune activation in the presence of PBMCs and monoclonal antibodies [20]. In the present study, the immunomodulatory component of the response is expected to arise mainly from the adenovirus–PBMC interaction, with chemotherapy and nintedanib acting synergistically to potentiate tumor cell death and antigen release.

The originality of our 3D system lies in the integration of multiple tumor microenvironment components—tumor spheroids, endothelial cells (HUVECs), and immune cells (PBMCs)—within a microfluidic perfusion-based platform, enabling simultaneous evaluation of cytotoxic, anti-angiogenic, and immunomodulatory effects under controlled dynamic conditions. The dynamic flow condition provides significant advantages over traditional static cultures. Continuous perfusion enhances nutrient and oxygen exchange, facilitates drug distribution, and supports the migration and interaction of PBMCs and endothelial cells with the tumor spheroids, closely resembling the in vivo microenvironment. Moreover, it allows a more realistic simulation of systemic drug exposure and immune cell trafficking, which are not adequately captured in static systems. Collectively, these features make the dynamic 3D co-culture a more physiologically relevant and translationally meaningful platform for preclinical testing of combination immuno-chemovirotherapies.

This dynamic tumor-on-a-chip approach offers a more translationally relevant platform for optimizing combination therapies, bridging the gap between in vitro and in vivo models in ovarian cancer research [20, 53]. Human ovarian cancer spheroids based on A2780 and OVCAR-3 cells were established using the hanging-drop method (Supplementary Fig. 5a), which differs from the previous one used in the static three-dimensional cell culture model. While the first method employed was straightforward and effective, it had limitations for the set-up of the newly established microfluidic system. Thus, a cell-number screening was performed to determine the optimal number of cells needed to form spheroids within a desired size and morphology (A2780: 600 cells/well; OVCAR-3: 1000 cells/well) (Supplementary Fig. 5b).

**Fig. 6:**
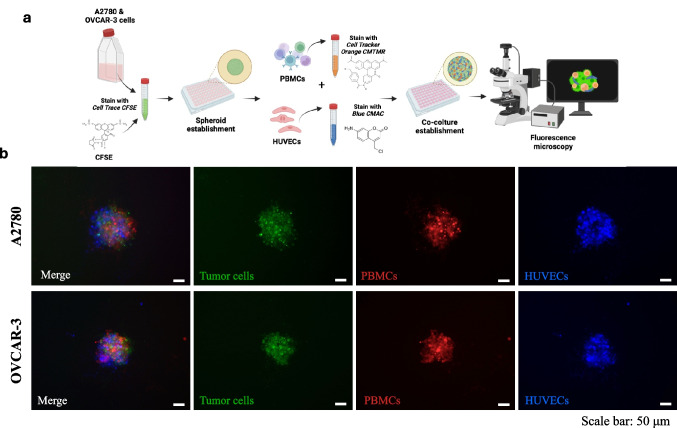
3D coculture establishment and characterization through fluorescent microscopy. **a**-**b** A2780 and OVCAR-3 cells were stained with Cell Trace CFSE green and seeded at a density of 400 cells/well and 1000 cells/well, respectively in 96 well-plate and centrifuged at 1000 rpm for 5 min to form spheroids. On day 6, PBMCs stained with CellTracker orange and HUVECs stained with Blue CMAC were added to tumor spheroids in a ratio 1:5. After 24 h, images were captured using a fluorescence microscope using 10 × objective. Images were assembled using ImageJ software

**Fig. 7:**
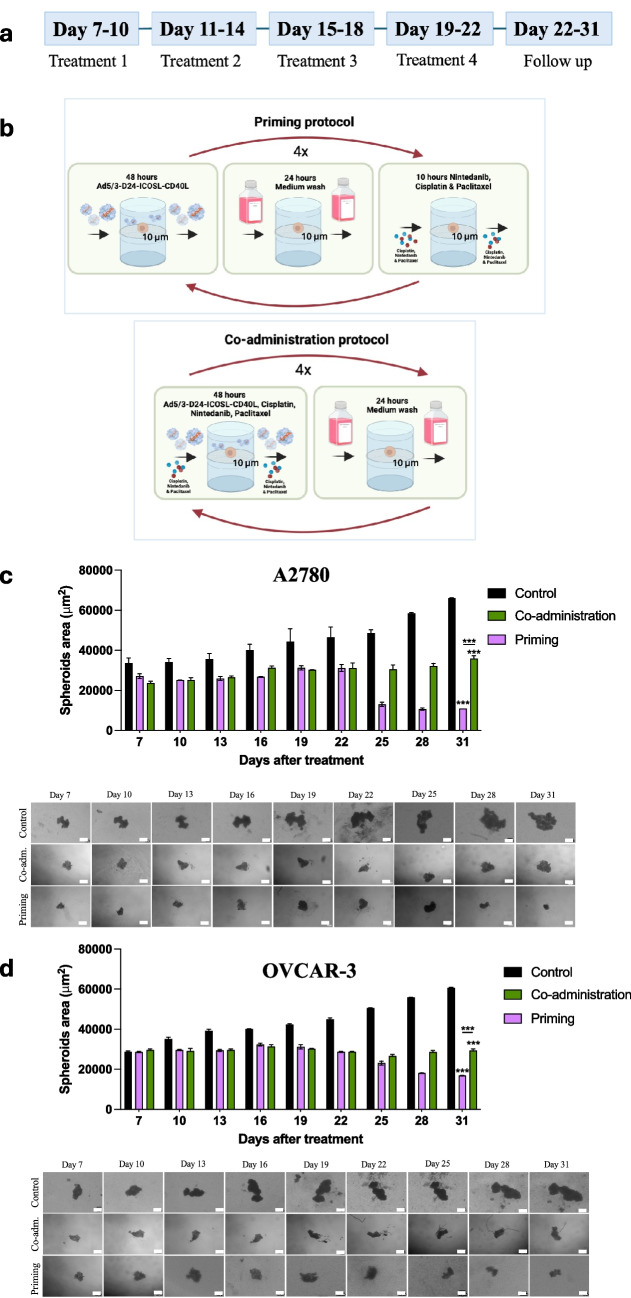
Assessment of anticancer efficacy of combination therapy on human ovarian cancer spheroids. **a-b** A2780 and OVCAR-3 cells were seeded at a density of 400 cells/well and 1000 cells/well, respectively in a 96-well plate with 1.5% agarose and centrifuged at 1000 rpm for 5 min to form spheroids. On day 6, PBMCs and HUVECs were added to the spheroids. The next day, spheroids were treated according to Table 2*.* Spheroids were examined microscopically every three days until day 31. Statistical analysis was performed using one-way ANOVA on day 31 (control vs priming: (***P ≤ 0.001; control vs co-administration: (***P ≤ 0.001; priming vs co-administration: (***P ≤ 0.001). **c-d** Spheroid size was measured on two dimensions every three days using Zeiss AxioVision software. Representative optic microscope pictures of the treated spheroids throughout the experiments. Scale bar: 50 μm

Before proceeding with the microfluidic platform analysis, it was essential to confirm the successful integration of immune components (PBMCs) and angiogenic factors (HUVECs) within the spheroids composed of A2780 and OVCAR-3 cells. To achieve this, fluorescence microscopy was employed to visualize and characterize the 3D coculture system (Fig. 6). Then, given that the microfluidic platform had only two available spots, we decided to focus on the analysis and comparison of both co-administration and priming protocol in both cell lines (Fig. 7b-c). This decision was made to explore the dynamic potential and comprehensive evaluation of these two experimental conditions [50]. The implementation of a perfusable, dual-chambered microfluidic system has revolutionized the ability to monitor spheroid responses in real-time under flow conditions that closely mimic systemic drug delivery. This innovation significantly enhances the translational relevance of our experimental model. By integrating perfusion-driven immune infiltration (via PBMCs) and vascular simulation (via HUVECs), this platform recapitulates the spatial and temporal complexity of the tumor microenvironment, offering a unique opportunity to evaluate the pharmacokinetic and pharmacodynamic interactions of combination therapies. This unique setup allows for the thorough evaluation of pharmacokinetic and pharmacodynamic interactions in combination therapies.

In order to facilitate immune cell infiltration, drug diffusion, and physiologically relevant tumor-stroma interactions in the microfluidic system, a 10 µm semi-permeable polyethylene terephthalate (PET) membrane was selected. This pore size allows PBMC migration, mimicking immune infiltration crucial for evaluating oncolytic virotherapy [55]. Additionally, it supports the diffusion of cytokines, chemokines, and chemotherapeutics (cisplatin, paclitaxel, nintedanib) while maintaining tumor spheroid integrity, preventing uncontrolled cell dispersal [12, 32]. The membrane also ensures mechanical stability and fluid perfusion, replicating vascular-tumor interactions and systemic drug delivery [56]. Compatible with standard incubator conditions, it enhances the physiological relevance of the model, making it an optimal platform for studying combination therapies in ovarian cancer [57]. Moreover, the system's semi-permeable membrane plays a crucial role in facilitating controlled interactions between compartments, thereby optimizing drug diffusion and immune cell migration while preserving the integrity of the spheroids.

Our findings demonstrate that in the ovarian cancer-derived 3D coculture models integrated within a dynamic microfluidic platform, the priming strategy—where Ad5/3-D24-ICOSL-CD40L was administered 48 h prior to the triple-drug regimen—resulted in a markedly enhanced antitumor response, as evidenced by significantly smaller spheroid areas (A2780: 10,895 µm^2^; OVCAR-3: 16,845 µm^2^) compared to the co-administration protocol (A2780: 35,895 µm^2^; OVCAR-3: 29,395 µm^2^). This highlights the critical role of treatment schedule in multimodal regimens (Fig. 7c-d). The improved therapeutic outcome is likely attributable to the immune-preconditioning effect of the oncolytic virus, which enables optimal replication and immune activation before the onset of chemotherapy-induced cytotoxic stress, a condition known to hinder viral efficacy. This immune-preconditioning effect is supported by clinical studies using ONCOS-102, another genetically modified oncolytic adenovirus engineered to express granulocyte–macrophage colony-stimulating factor (GM-CSF) to enhance anti-tumor immunity [21–24]. ONCOS-102 has demonstrated increased tumor infiltration by cytotoxic T cells, resulting in enhanced immune-mediated tumor destruction in ovarian cancer and other solid tumors. Similarly, our findings suggest that ICOSL and CD40L expression in Ad5/3-D24-ICOSL-CD40L may provide an additional immune-stimulatory benefit.

Interestingly, our microfluidic model has elucidated the distinct effects of oncolytic virotherapy (OV) on tumor-immune crosstalk, as evidenced by increased ATP release and calreticulin exposure—both indicative of immunogenic cell death (ICD) (Fig. 4). These findings are consistent with previous studies that demonstrate how oncolytic virotherapy enhances ICD, promotes tumor antigen presentation, and facilitates adaptive immune activation [46, 47]. The ability of OV to stimulate a pro-inflammatory tumor microenvironment (TME) fosters greater immune cell infiltration and extends therapeutic efficacy, marking it as a pivotal element in combination therapy regimens.

The observed benefits of viral priming align with earlier findings on TILT-123 and VALO-D102, which underscore the significance of OV-mediated immune priming in enhancing subsequent immunotherapeutic efficacy, especially in conjunction with checkpoint blockade or T-cell co-stimulation [53, 54]. Looking to the future, integrating this microfluidic tumor-on-a-chip system with advanced drug delivery technologies—such as nanoparticle carriers or biomaterial scaffolds—holds promise for improving targeted biodistribution and retention within tumor tissues.

Furthermore, the use of a perfusable, dual-chambered microfluidic system enabled real-time monitoring of spheroid response under flow conditions mimicking systemic drug delivery, thereby enhancing the translational relevance of the model. Additionally, the system's semi-permeable membrane facilitated controlled crosstalk between compartments, optimizing drug diffusion and immune cell migration without compromising spheroid integrity the use of microfluidic technology allows for dynamic monitoring of tumor responses, revealing that the priming strategy not only leads to sustained tumor reduction but also mitigates the rebound effect often seen in chemotherapy-treated models. This suggests that oncolytic virotherapy may extend the therapeutic window and prevent tumor relapse, addressing a critical limitation of current ovarian cancer treatments. The results of this study bolster the growing evidence that sequential OV priming enhances antitumor activity, particularly when paired with immunotherapeutic agents such as checkpoint inhibitors or T-cell activators, as demonstrated with TILT-123 and VALO-D102 [54, 55]. Collectively, these findings highlight the potential of oncolytic virotherapy-based regimens to counteract tumor resistance mechanisms and improve clinical outcomes for patients facing refractory or relapsed ovarian cancer.

## Conclusion

In this study, we demonstrated that the adenovirus Ad5/3-D24-ICOSL-CD40L efficiently infects and oncolyses ovarian cancer cells while exhibiting potent immunomodulatory effects. This reinforces its potential as a therapeutic immune activator for clinical translation. The ability of this oncolytic adenovirus to enhance tumor immunogenicity and induce immunogenic cell death suggests that it may be particularly effective when integrated into combination regimens that activate the immune system. Furthermore, we developed a microfluidic 3D tumor model that provides a physiologically relevant platform for assessing the efficacy of complex combination therapies. Unlike traditional 2D or static 3D culture systems, our model incorporates continuous fluid dynamics, immune cell infiltration, and endothelial interactions, closely mimicking the tumor microenvironment and enabling simulation of systemic drug delivery. Our findings showed that priming with Ad5/3-D24-ICOSL-CD40L prior to chemotherapy resulted in enhanced viral replication, improved treatment efficacy, and reduced tumor rebound effects, outperforming conventional co-administration strategies. This underscores the potential of integrating oncolytic virotherapy with chemotherapy and targeted therapy to improve treatment outcomes in ovarian cancer patients, particularly those with chemoresistant or relapsed disease.

Moreover, the microfluidic 3D tumor model serves as a powerful preclinical platform for refining combination therapies, optimizing treatment sequencing, and predicting patient responses. By closely mimicking the tumor microenvironment and dynamic drug interactions, this system bridges the gap between in vitro studies and clinical translation, ultimately accelerating the development of more effective, precision-based cancer therapeutic strategies. In the future, it could also assess the safety, distribution, and efficacy of advanced nanomedicine-based therapeutics, offering a valuable tool for predicting in vivo performance and pharmacodynamics in complex tumor settings.

Despite these promising results, further investigations are essential to validate our findings in vivo, particularly using patient-derived xenograft (PDX), humanized models, and organoid models, to fully elucidate the therapeutic potential. Additionally, optimizing combination strategies—including ideal dosing regimens, sequencing, and administration routes—will be critical for translating this approach into clinical settings. Future studies should also focus on identifying predictive biomarkers to stratify patients most likely to benefit from oncolytic virotherapy-based combination treatments, ensuring a more personalized and targeted therapeutic approach.

## Supplementary Information

Below is the link to the electronic supplementary material.

ESM 1 (DOXC 1.54 MB)

## Data Availability

The data generated for the current study are available from the corresponding author. The authors commit to providing data and materials supporting the findings and analyses in this paper upon reasonable request.
