# Supplementary material for: Dynamic 3D microfluidic platform for exploring combined targeted therapy, chemotherapy, and virotherapy delivery in ovarian cancer
Source: Drug Deliv Transl Res. 2025 Nov 3;16(8):2678–96. doi: 10.1007/s13346-025-01997-4 (PMC13346287; doi:10.1007/s13346-025-01997-4)
Supplement: Supplementary file 1 — (DOXC 1.54 MB) [file 13346_2025_1997_MOESM1_ESM.docx]

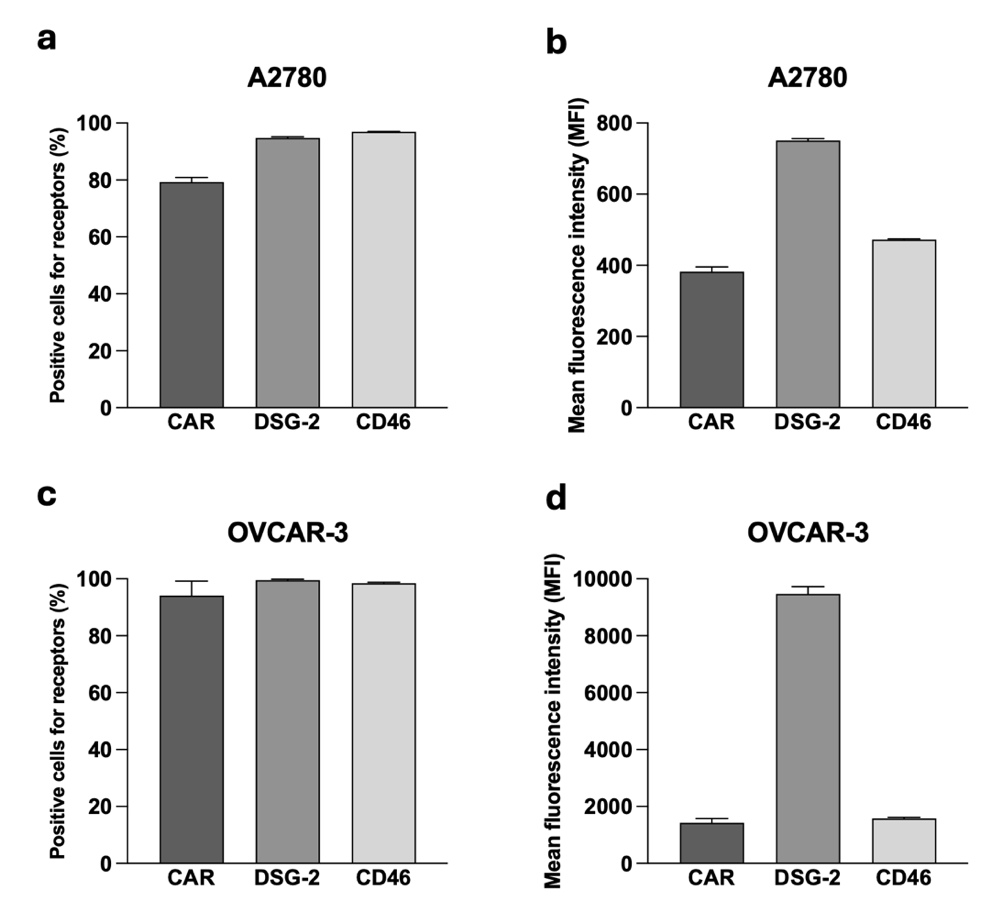


**Supplementary Fig. 1 Expression of CAR, DSG-2 and CD46 receptors on selected human ovarian cancer cells.** Adenoviral receptor’s expression levels were measured 24 hours after cell seeding. Data are expressed as percentage of positive cells **(a-c)** and mean fluorescence intensity **(b-d)** for CAR, DSG-2 and CD46 measured by flow cytometric analysis (BD FACSAria III, Becton Dickinson, USA) through specific antibody staining

**
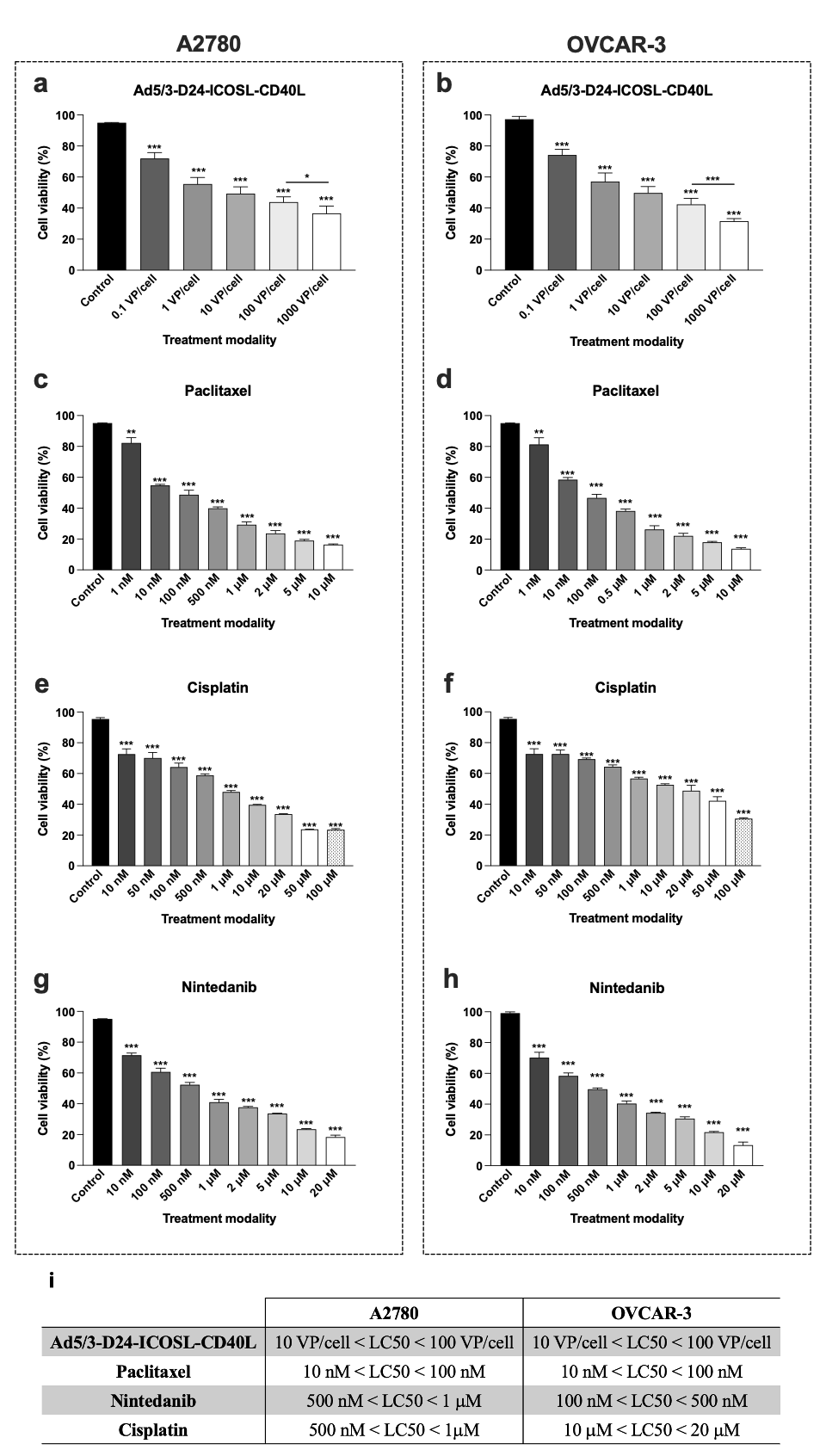
**

**Supplementary Fig. 2 Cell viability assay of antitumoral agents as monotherapies and LC50 evaluation on selected ovarian cancer cells**. Cell viability was evaluated on ovarian cancer cells 72 hours post-treatment with increasing concentrations of AdV5/3-D24-ICOSL-CD40L **(a-b)**, paclitaxel **(c-d)**, cisplatin **(e-f)**, and nintedanib **(g-h)**. Data are expressed as the percentage of viable cells, determined using the CellTiter 96 Aqueous One Solution Cell Proliferation Assay by measuring the absorbance at 490 nm with a 96-well plate spectrophotometer, INNO ™. Statistical analysis was performed using one-way ANOVA (**P ≤ 0.001, ***P ≤ 0.0001). **(i)** LC50 ranges for each antitumoral agent


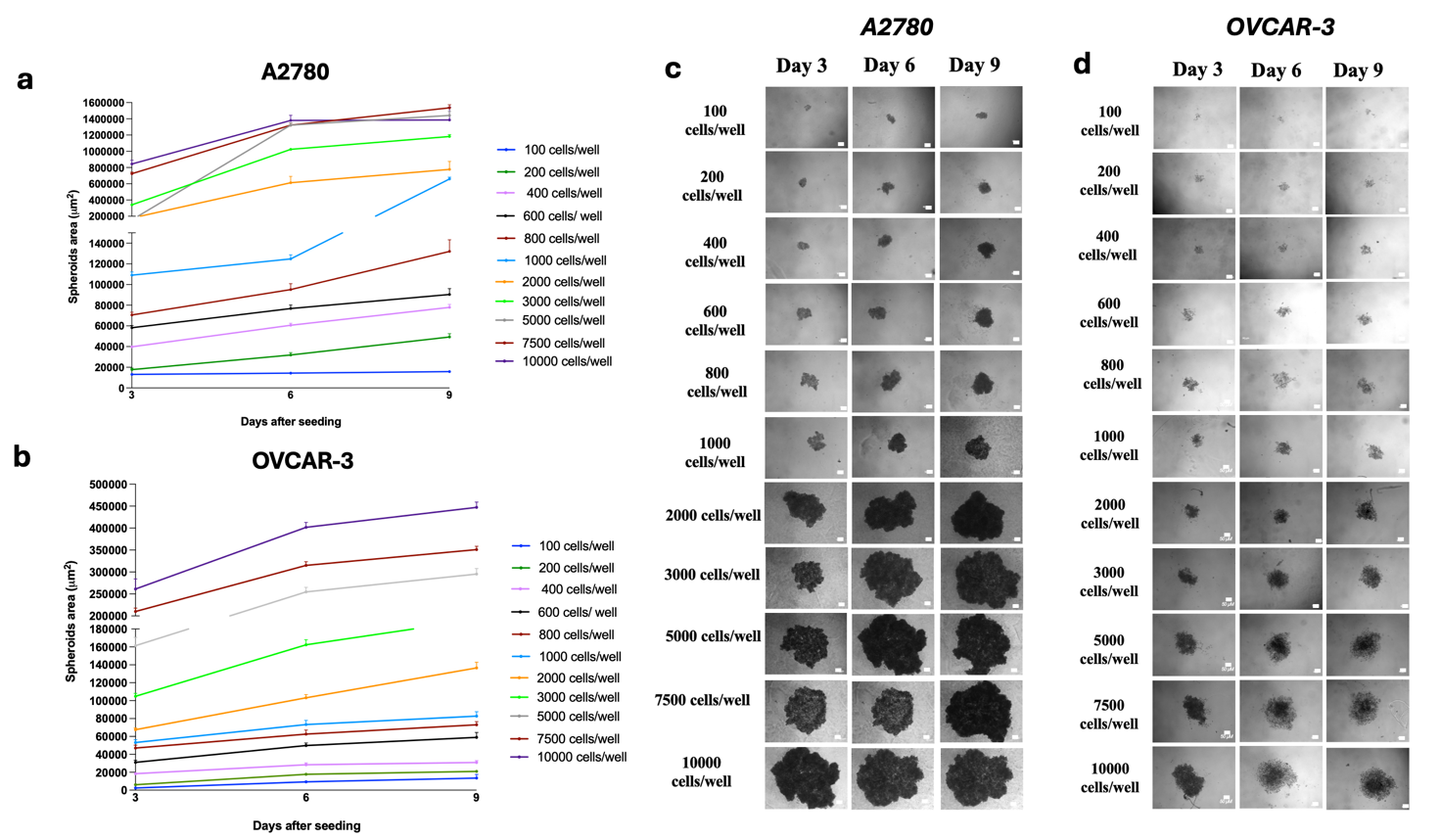


**Supplementary Fig. 3 Establishment of 3D ovarian cancer models. (a-b)** Ovarian cancer cell lines were seeded at different densities (100 – 200 – 400 – 600 – 800 – 1000 – 2000 – 3000 – 5000 – 7500 – 10000 cells/well) into 96-well plates with flat bottom previously coated with 1.5% of agarose solution. After seeding, the plates were centrifuged at 1000 rpm for 5 minutes. Morphology and growth were then monitored over time for 9 days. Every three days the cell culture medium was changed, and the spheroid area was registered. **(c-d)** Representative images showing the morphology of spheroids visualized by optic microscopy

**Supplementary Fig. 4 Dose screening of the antitumoral agents in 3D ovarian cancer models.** A2780 and OVCAR-3 spheroids were treated with multiple administration of Ad5/3-D24-ICOSL-CD40L **(a-b)**, paclitaxel **(c-d)**, nintedanib **(e-f)**, cisplatin **(g-h)**. Spheroid morphology (n=4/group) was checked daily overtime and spheroid areas were registered every three days. Statistical analysis was performed with one-way ANOVA (***P ≤ 0.001, **P≤0.01)

**
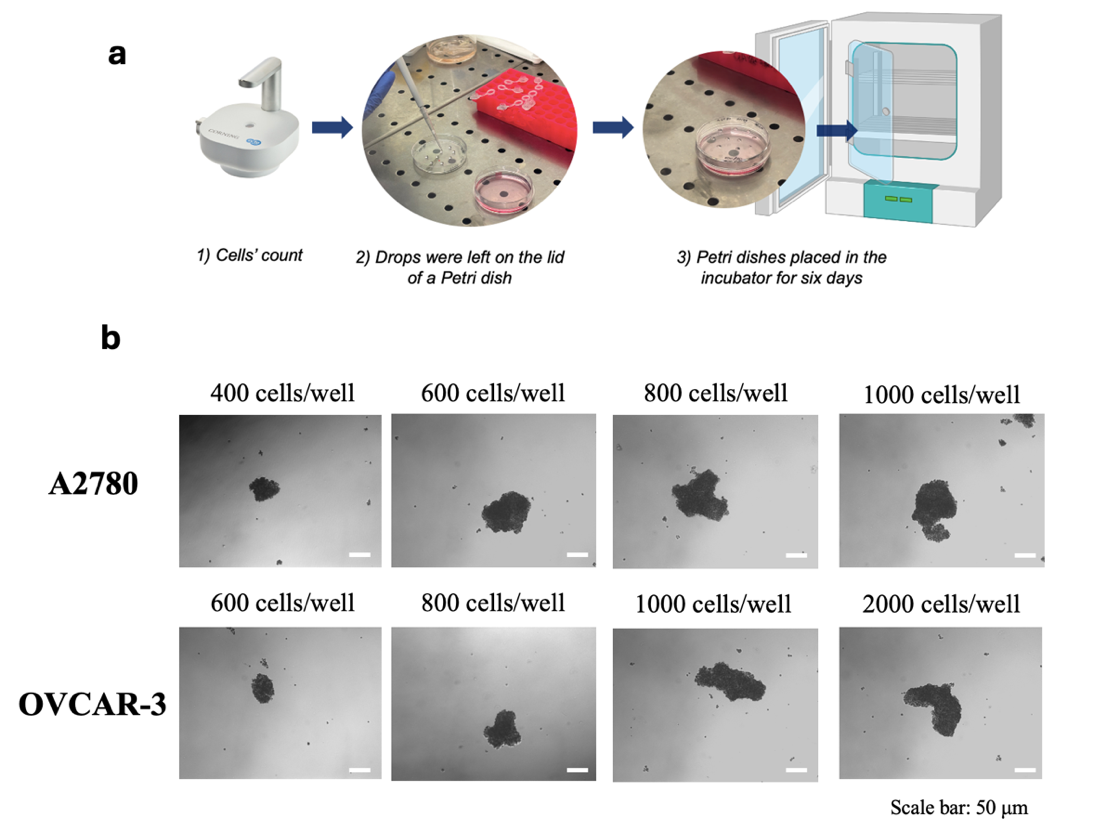
**

**Supplementary Fig. 5 Establishment of ovarian cancer spheroids by hanging-drop method.** **(a)** Schematic representation of the hanging drop method used for spheroid formation. **(b)** Representative brightfield images of spheroids formed by A2780 and OVCAR-3 ovarian cancer cell lines at different seeding densities. Scale bar: 50 µm
